# Electroacupuncture Mitigates Skeletal Muscular Lipid Metabolism Disorder Related to High-Fat-Diet Induced Insulin Resistance through the AMPK/ACC Signaling Pathway

**DOI:** 10.1155/2018/7925842

**Published:** 2018-11-07

**Authors:** Zhixing Li, Danchun Lan, Haihua Zhang, Hongtao Zhang, Xiaozhuan Chen, Jian Sun

**Affiliations:** ^1^Department of Soft Tissue Traumatology, Fourth Affiliated Hospital of Guangzhou University of Chinese Medicine, Shenzhen 518033, China; ^2^Department of Acu-Moxibustion, Foshan Hospital of Traditional Chinese Medicine, Foshan, Foshan 528000, China; ^3^Massage Department, Fourth Affiliated Hospital of Guangzhou University of Chinese Medicine, Shenzhen 518033, China; ^4^Traditional Therapy Department of Fangchun, Second Affiliated Hospital of Guangzhou University of Chinese Medicine, Guangzhou 510000, China

## Abstract

The aim of this work is to investigate the effect of electroacupuncture (EA) on insulin sensitivity in high-fat diet (HFD) induced insulin resistance (IR) rats and to evaluate expression of AMPK/ACC signaling components. Thirty-two male Sprague-Dawley rats were randomized into control group, HFD group, HFD+Pi (oral gavage of pioglitazone) group, and HFD+EA group. Acupuncture was subcutaneously applied to Zusanli (ST40) and Sanyinjiao (SP6). For Zusanli (ST40) and Sanyinjiao (SP6), needles were connected to an electroacupuncture (EA) apparatus. Fasting plasma glucose was measured by glucose oxidase method. Plasma fasting insulin (FINS) and adiponectin (ADP) were determined by ELISA. Triglyceride (TG) and cholesterol (TC) were determined by Gpo-pap. Proteins of adiponectin receptor 1 (adipoR1), AMP-activated Protein Kinase (AMPK), and acetyl-CoA carboxylase (ACC) were determined by Western blot, respectively. Compared with the control group, HFD group exhibits increased levels of FPG, FINS, and homeostatic model assessment of insulin resistance (HOMA-IR) and decreased level of ADP and insulin sensitivity index (ISI). These changes were reversed by both EA and pioglitazone. Proteins of adipoR1 and AMPK were decreased, while ACC were increased in HFD group compared to control group. Proteins of these molecules were restored back to normal levels upon EA and pioglitazone. EA can improve the insulin sensitivity of insulin resistance rats; the positive regulation of the AMPK/ACC pathway in the skeletal muscle may be a possible mechanism of EA in the treatment of IR.

## 1. Introduction

At the turn of this century 171 million individuals were estimated to have diabetes, and this is expected to increase to 366 million by 2030[1]. Type 2 diabetes mellitus (T2DM) may lead to severe complications and huge financial burden against patients [2]. The prolonged asymptomatic phase of T2DM may last years, during which time unmanaged elevated blood glucose could lead to serious and irreversible micro- and macrovascular complications including neuropathy, nephropathy, retinopathy, coronary artery disease, stroke, and peripheral vascular disease [3, 4]. Although the underlying cause has not been fully clarified, it is generally attributed to insulin resistance (IR), which precedes formal diagnosis of T2DM for years [5]. It has been well established that both insulin resistance and obesity play an important role in the pathogenesis of T2DM [6] and therefore their amelioration is a critical clinical goal. As we know disordered lipid metabolism is the characteristic of obesity and IR [7]. Consequently many treatment strategies are designed to promote insulin sensitivity by regulating lipid metabolism.

Obesity is associated with an increased risk of developing IR and T2DM. In obese individuals, adipose tissue modulates metabolism by releasing increased amounts of nonesterified fatty acids (NEFAs), glycerol, hormones—including leptin and adiponectin—and proinflammatory cytokines, and other factors that are involved in the development of insulin resistance [8]. Previous studies have suggested that insulin resistance develops secondary to diminished fat oxidation and resultant accumulation of cytosolic lipid molecules that impair insulin signaling [9]. The skeletal muscle is recognized as a classical insulin sensitive organ as well as a key regulator of glucose uptake [10]. Metabolic inflexibility in fat oxidation is a component of skeletal muscle insulin resistance in obesity and T2DM [11]. Increased NEFAs levels that exceed the storage of adipose tissue can result in lipotoxicity. The accumulation of fat in tissues not suited for lipid storage has deleterious consequences on skeletal muscle function, leading to cellular damage [12]. Loss of skeletal muscle function is likely to eventually contribute to insulin resistance.

Increased NEFA delivery or decreased intracellular metabolism of fatty acids results in an increase in the intracellular content of fatty acid metabolites such as diacylglycerol, fatty acyl-coenzyme A, and ceramides, which, in turn, activate a serine/threonine kinase cascade leading to serine/threonine phosphorylation of insulin receptor substrate-1 (IRS-1) and insulin receptor substrate-2 (IRS-2), and a reduced ability of these molecules to activate PI3K, which is associated with altered rates of insulin-stimulated glucose uptake and transport [13, 14]. By contrast, adiponectin acts as an insulin sensitizer, stimulating fatty acid oxidation in an AMP-activated protein kinase (AMPK), and peroxisome proliferator activated receptor-*α*- (PPAR-*α*-) dependent manner [8]. Thiazolidinediones (TZDs) are a class of antidiabetic drugs that increase systemic insulin sensitivity in tissues of animal models [15] and humans with T2DM and the metabolic syndrome [16]. Pioglitazone is currently used to treat patients with T2DM which has been shown to activate AMPK activity in cultured cells [17]. Pharmacological agents are clinically available; however, they carry risks of serious complications and high costs [18]. Eletroacupuncture (EA) has historically been used as an adjuvant therapy to treat IR [19]. This therapy appears to be effective in decreasing TC, TG, and LDL-C levels, increasing insulin levels and improving glucose tolerance [20, 21]. The capacity for EA to improve lipid metabolism and increase insulin sensitivity has been demonstrated; however, the effects of EA intervention on the AMPK and its associated proteins in IR cases remain largely elusive.

Therefore, the goal of this study is to elucidate the EA effect on AMPK/ACC pathway proteins and further clarify the mechanism of EA in improving lipid metabolism in skeletal muscle of IR cases.

## 2. Materials and Methods

### 2.1. Experimental Animals

Male Sprague-Dawley rats (n=32, 8 weeks, clean grade, weighing 200.3 ± 23.3 g) were purchased from the Experimental Animal Center of Guangzhou University of Chinese Medicine (certificate number: SCXK (Guangzhou) 2013-0002). Animals were maintained in the Experimental Animal Center of Traditional Chinese Medicine Hospital of Guangdong Province. The animals were reared in separate cages in a room under controlled light, relative humidity (50%), and temperature (20±2°C) conditions. All animals had free access to basic feed and tap water. All animal experimental procedures were reviewed and approved by the Laboratory Animals Ethics Committee at Second Affiliated Hospital of Guangzhou University of Chinese Medicine Hospital (reference no. 2014016) and were conducted in accordance with local guidelines for animal welfare consistent with the National Research Council's ‘Guide for the Care and Use of Laboratory Animals' (National Academies Press, Washington, DC, USA). All efforts were made to minimise the number of animals used and their suffering.

### 2.2. Animal Grouping and Intervention

According to a random number table, the rats were allocated to a negative control group (control group, n=8) or a IR model group (n=24). Control group was fed with a regular diet (RD) consisting of 21% protein, 11% fat and 68% Carbohydrates. IR model group was fed a high fat diet (HFD) comprising 26.2% crude protein, 34.9% total fat, and 5% crude fiber. Twelve weeks later, the remaining 24 IR rats were subdivided into three groups according to a random number table (n=8 each): (1) an IR group remained untreated; (2) an IR + Pi group that received oral gavage of pioglitazone (10mg/kg/day); and (3) an IR + EA group that received EA only. Rats in the IR + EA group were physically restrained during EA and rats in the other three groups were restrained in the same way without receiving acupuncture to control for any effect of handling on study outcomes. Venous blood of rats was collected for fasting insulin, fasting plasma glucose, triglyceride, cholesterol, and HOMA-IR index measurement after treatment. All rats were anesthetized by intraperitoneal injection of sodium pentobarbital (50mg/kg) and were sacrificed by cervical dislocation for histopathological examination on the skeletal muscle. Skeletal muscles peeled off from quadriceps femoris were stored at −80°C for the following Western blotting.

### 2.3. EA Therapy

EA was administered every day to rats in the IR + EA group only, seven days a week for 2 consecutive weeks. EA treatment was performed at classical acupuncture points, namely ST40 (*Fenglong*) and SP6 (*Sanyinjiao*) [22]. A pair of stainless steel needles (0.35 mm diameter, 25 mm length; GB2024-1994, Huatuo, Suzhou, China) were connected to the output terminals of an EA apparatus (model no. G6805-2A, Shanghai Medical Electronic Apparatus Company, China) and EA was applied using alternating strings of dense-sparse waves at alternating frequencies (50 Hz for 1.05s and 2 Hz for 2.85s, pulse, pulse width 0.5 ms). The intensity was adjusted to induce slight twitching of the hindlimbs (<1 mA) and each treatment lasted 20 min. All rats of the four groups were manipulated by hands of researchers.

### 2.4. Plasma and Serum Analyses

All rats were fasted for 12 hours after the last acupuncture. On the second morning, blood samples from the orbital venous sinus were collected. Samples were measured for fasting insulin (FINS), fasting plasma glucose (FPG), triglyceride (TG), cholesterol (TC), and adiponectin (ADP). FPG was measured by glucose oxidase method. FINS and ADP were determined by ELISA method (catalogue no. 10-1250-01, Mercodia AB, Uppsala, Sweden; catalogue no. AG-45A-0005, Adipogen Corporation, San Diego, USA). TG and TC were determined by Gpo-pap method (catalogue no. A110, A111, respectively; Nanjing Jiancheng Bioengineering Institute, Nanjing, China). All of above methods were performed according to the instructions from the manufacturer. Additionally, the following formula was used to calculate homeostasis model assessment-estimated insulin resistance (HOMA-IR)= FINS (IU/L) × FPG (mmol/L)/22.5[23]. Insulin sensitivity was determined by the insulin sensitivity index (ISI)= −In (FINS × FPG) [24].

### 2.5. Ultrastructural Examination by Transmission Electron Microscopy

4% paraformaldehyde fixed skeletal muscle of quadriceps femoris samples (1mm ×1mm) of control, IR, IR + EA, IR + pioglitazone groups was collected and fixed in 2.5% glutaraldehyde in 0.1 mol/L sodium phosphate buffer (pH 7.4) overnight at 4°C. After dehydration with a graded ethanol series, the sample was embedded in Epon812 and sectioned using a Leica EM UC6 ultramicrotome (Leica Co., Vienna, Austria). Samples were viewed and photographed using Transmission Electron Microscopy (TEM) (JEM-1200EX, JEDL, Japan).

### 2.6. Western Blotting

Skeletal muscle were peeled off from quadriceps femoris and washed twice with ice-cold PBS (PH 7.4) and then homogenized with RIPA buffer (Cell Signaling Technologies, Beverly, MA) containing protease inhibitor cocktail (Roche, Basel, Switzerland). Tissue lysates were then centrifuged at 12,000 g for 5 min at 4°C. Protein concentration was determined using BCA (Thermo, Waltham, MA), and 30 ug of total proteins was taken for 10% SDS polyacrylamide gel electrophoresis. The separated proteins were then transferred on to 0.2 pore polyvinylidene difluoride (PVDF) membranes (Millipore, Bedford, MA) using tank transfer system (Bio-Rad, Hercules, CA). The nonspecific binding of antibodies was blocked with 5% nonfat dried milk in TBST buffer (0.1%Tween 20 in PBS). The membrane was then transferred to the hybrid bag and the first antibody, namely, adipoR1 (no. sc - 46748; Santa Cruz Biotechnology, Dallas, TX, USA), AMPK, ACC, and GAPDH (catalogue no. 5831, 3676 and 5174, respectively; Cell Signaling Technology Company, Danvers, MA, USA), was added at 1:1000 dilution. The bag was then sealed and maintained at 4°C overnight. Subsequently, the second antibody (no. 7074; Cell Signaling Technology Company, Danvers, MA, USA) was added, and the membrane was incubated for 1 hour at 37°C and washed. After reacting with enhanced chemiluminescence (ECL) substrate for 1–3 min, the light signal was captured by Chemidoc gel imaging system (Bio-Rad, Hercules, CA), and the image was analyzed by Quantity One 1D Analysis Software (Bio-Rad, Hercules, CA).

### 2.7. Statistical Analysis

The statistical analysis was performed using the SPSS software, version 20.0 (SPSS, IBM, Armonk, NY, USA), and the data were expressed as the mean ± standard deviation. A one-way ANOVA was used after the normal distribution and homogeneity of variance were confirmed. For the nonnormally distributed data or for data with heterogeneous variance, a nonparametric test was used. The LSD method was applied for pairwise comparisons of the Western blot results. Statistical significance was set to p < 0.05 and high statistical significance was set to p < 0.01.

## 3. Results

### 3.1. Hematological Assessment Results

**Figure 1:**
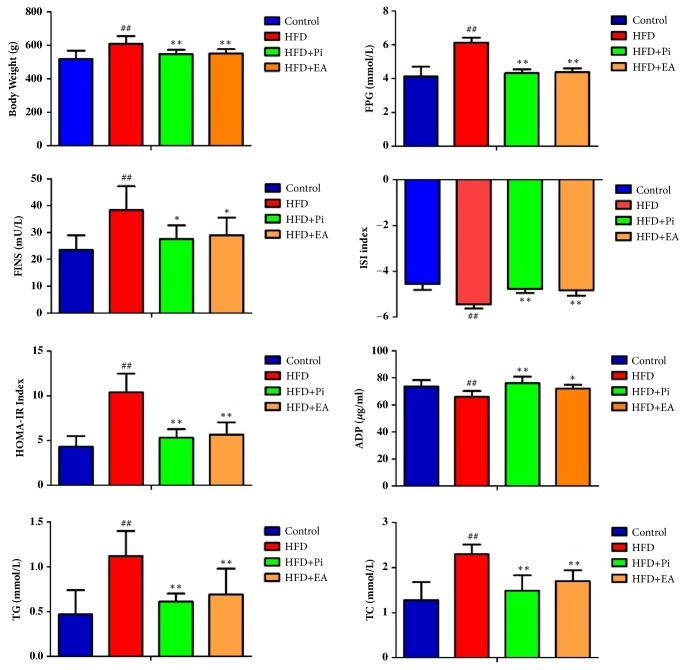
Comparison of the body weight, fasting plasma concentrations of glucose (FPG), insulin (FINS), insulin sensitivity index (ISI), adiponectin (ADP), triglyceride (TG), cholesterol (TC), and homeostatic model assessment of insulin resistance (HOMA-IR) index in each group. Values represent means ± SD (n = 8 each group). ^#^* p* < 0.05,^ ##^* p* < 0.01 compared with the control group and ^*∗*^*p* < 0.05, ^*∗∗*^*p* < 0.01 compared with the HFD group.

As shown in Figure 1, the level of body weight, plasma FPG, FINS, TG, TC, and HOMA-IR in the HFD group was significantly increased, while the level of ADP and ISI was significantly decreased in the 8 successfully modeled rats as compared with the control group (*p *< 0.01). After EA and pioglitazone treatment, the levels of body weight, plasma FPG, FINS, TG, TC, and HOMA-IR in the HFD + Pi and HFD + EA group were significantly decreased, while the levels of ADP and ISI were significantly increased as compared with the HFD group (*p *< 0.05,* p *< 0.01). There were no significant differences observed between HFD+Pi and HFD+EA groups (*p* > 0.05).

### 3.2. Histopathology

**Figure 2:**
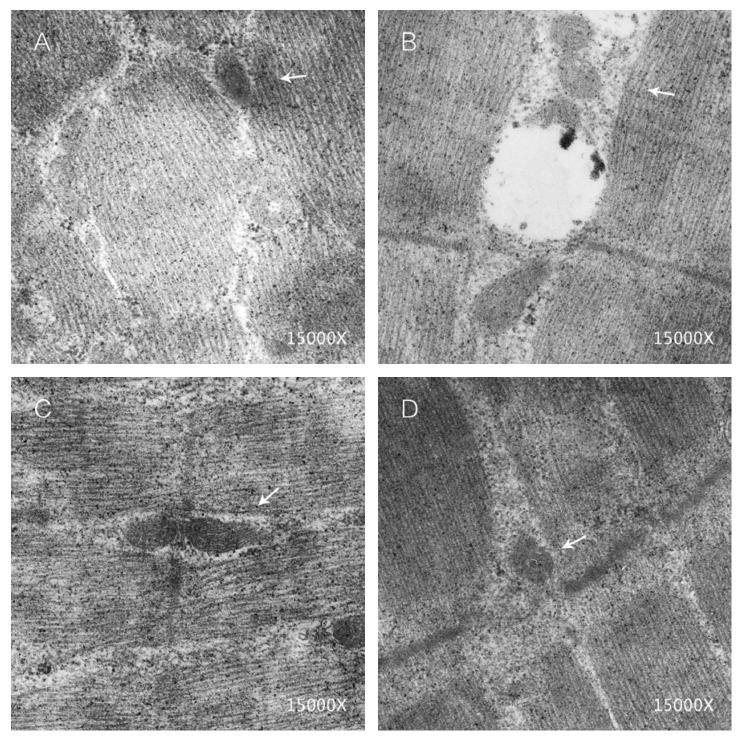
Transmission electron microscope image showing ultrastructural features of skeletal muscle of rats from all groups. A: control group, normal mitochondria and fiber structure. B: HFD group, fat droplet accumulation, damaged, swollen, or fused mitochondria, and disordered fiber arrangement or dysfunctional fiber structure. C: HFD + Pi group, the disordered fiber arrangement improved and less damaged or fusion mitochondria. D: HFD + EA group, mitochondria and fiber structure reversed to normal. White arrows indicate the region of mitochondria.

The quality of functional status of mitochondria in skeletal muscle was examined and evaluated by transmission electron microscope. Compared with the control group, many vacuoles with different sizes and subsarcolemmal accumulation of enlarged and abnormally shaped or swollen mitochondria were observed in HFD group. One of the hallmarks for the disordered lipid metabolism of skeletal muscle is the high population of damaged or swollen mitochondria and disordered fiber arrangement or dysfunctional fiber structure in skeletal muscle. The normal arrangement of mitochondria and apparently less vacuoles were observed in skeletal muscle from HFD + Pi and HFD + EA groups (Figure 2).

### 3.3. Skeletal Muscle Expression of AdipoR1, AMPK, and ACC

**Figure 3:**
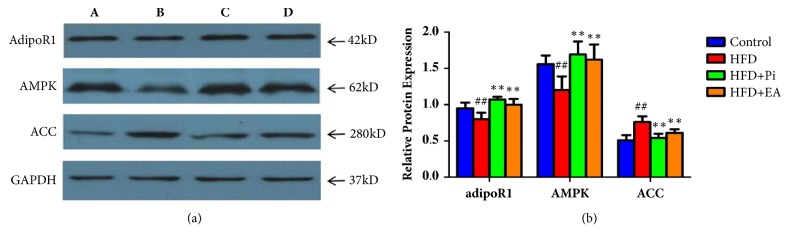
Comparison of the protein expression of adipoR1, AMPK, and ACC in skeletal muscle in each group. A: control group, B: HFD group, C: HFD+ Pi group, and D: HFD+EA group. GAPDH was used as an internal control. Data are shown as mean ± SD (n = 8 each group). ^#^* p *< 0.05, ^##^* p* < 0.01 compared with the control group and ^*∗*^*p* < 0.05, ^*∗∗*^*p* < 0.01 compared with the HFD group.

Figure 3 illustrates the results from Western blotting for levels of skeletal muscle adipoR1, AMPK, and ACC protein expression in the four groups after intervention. Expression of adipoR1 and AMPK was significantly decreased, while ACC was significantly increased in the HFD group compared with those of the control group (*p *< 0.05,* p *< 0.01). The HFD + Pi and HFD + EA group showed increased expression of both adipoR1 and AMPK and decreased expression of ACC compared with those of the HFD group (all* p* < 0.01). No significant differences were obtained between the HFD + Pi and HFD + EA group (*p* > 0.05).

## 4. Discussion

This study demonstrated that EA intervention improved insulin resistance in HFD rats. Insulin sensitivity as measured by HOMA-IR index and ISI got worse in HFD rats after 12 weeks of HFD. This was consistent with previous reports that HFD rats developed insulin resistance and had higher plasma free fatty acids (FFAs) and triglycerides [25]. In the HFD model, decreased levels of adiponectin receptor 1 (adipoR1) and AMP-activated Protein Kinase (AMPK) were observed in skeletal muscle. In addition, acetyl-CoA carboxylase (ACC), the downstream mediator of AMPK, increased in the HFD model. Our research showed that two-week EA stimulation reversed blood glucose and insulin resistance in the HFD model to normal.

It is believed that EA may be used to prevent diabetes and metabolic syndrome since it can ameliorate insulin sensitivity. However, the underlying mechanism of action remains uncertain. Obesity is commonly associated with T2DM, coronary artery disease, and hypertension, and the coexistence of these diseases has been termed the metabolic syndrome [26]. Insulin resistance is a key feature of metabolic syndrome. Any defects in the insulin signaling cascade can cause insulin resistance. It is known that this pathway is impaired progressively through alterations in the protein levels and activities of the signaling molecules, enzymes, and transcription factors in insulin resistance caused by increased adiposity [27]. White adipose tissue (WAT) is a major energy storage and is important for energy homeostasis, which stores energy in the form of triglycerides during nutritional abundance and releases it as FFAs during nutritional deprivation [28, 29]. Adiponectin, an adipokine, secreted by WAT has recently attracted much attention because of its antidiabetic and antiatherogenic effects and is expected to be a novel therapeutic factor for diabetes and the metabolic syndrome [30]. Indeed, a decrease in the circulating levels of adiponectin has shown to contribute to the development of insulin resistance and the metabolic syndrome [31]. In the present study, EA increased the level of adiponectin in the IR rats, indicating that EA may improving insulin resistance through regulating lipid metabolism. TZDs are known to improve systemic insulin sensitivity in animal models of obesity-linked insulin resistance and diabetes by enhancing glucose disposal in skeletal muscle and suppressing gluconeogenesis in the liver [32]. Plasma adiponectin levels have been shown to be upregulated by pioglitazone [33].

It is known that the skeletal muscle is one of the most susceptible areas to insulin resistance. Adiponectin receptor 1 (adipoR1) is abundantly expressed in skeletal muscle which serve as receptor for adiponectin. AdipoR1 expression in the skeletal muscle of T2DM patients has been reported to be decreased [34]. Our results showed that insulin resistance decreases not only plasma adiponectin levels but also adipoR1 expression which is consistent with those of previous report [33]. Moreover, adipoR1 expression is positively correlated with plasma triglyceride and cholesterol concentrations [35]. With EA therapy, adipoR1 expression was increased. Importantly, adiponectin induces extracellular Ca(2+) influx by adipoR1, which was necessary for subsequent activation AMPK [36]. Furthermore, with respected to the molecular mechanisms underlying of adiponectin, previous study found that adiponectin could enhance muscle fat oxidation and glucose transport via AMPK activation and acetyl-CoA carboxylase inhibition [37].

AMPK represents an energy sensor and metabolic regulator that responds to hormone and nutrition status in vivo and exerts a regulatory effect in the hypothalamus and multiple peripheral tissues [38]. Activated AMPK stimulates glucose uptake and lipid oxidation in peripheral tissues [39, 40]. Its activity can be upregulated in starving or fasting conditions and downregulated by satiety or a wide array of factors, such as insulin, leptin, and glucose [41, 42]. The important role of AMPK in control of food intake may be mediated by malonyl-CoA, a downstream target of acetyl-CoA carboxylase (ACC) which is a substrate of AMPK [43]. Anabolic pathways including fatty acid synthesis, transcription of lipogenic enzymes, triglyceride synthesis, and cholesterol synthesis are invariably inhibited by AMPK/ACC pathway [44]. Fatty acid oxidation in skeletal muscle involves a rate-controlling step that is regulated by carnitine palmitoyltransferase 1 (CPT1). CPT1 transfers long-chain acyl-CoA into the mitochondria, and this process is inhibited allosterically by malonyl-CoA, synthesized from acetyl-CoA with ACC acting as a rate-limiting enzyme. AMPK directly phosphorylates and inactivates ACC, followed by decreased level of malonyl-CoA and increased CPT1 activity, thereby driving the entry of long-chain acyl-CoA into the mitochondria for b-oxidation to restore energy balance [45]. Our results showed that the postreceptor signal transduction was inhibited by high-fat-diet, with downregulated AMPK and upregulated ACC. However, we found that both EA and pioglitazone reversed these inhibitions.

Although the present study has shown that electroacupuncture alters expression of AMPK/ACC signaling pathway related molecules and enhances insulin sensitivity in a rat model of IR, there are some details which are expected to improve. First, phosphorylated-AMPK and phosphorylated-ACC will be measured to demonstrate better about the activation of the AMPK/ACC signaling pathway in the next study. Second, specific inhibition of AMPK can be used as a negative control to explain the effect of electroacupuncture on AMPK biological function more objectively. Finally, the acupuncture protocol can be changed to every other day and last for 4 weeks, which can ensure the acupuncture stimulation and be convenient for both patients and acupuncturists to follow.

## 5. Conclusion

The present study suggests that EA increase insulin sensitivity via modulating secretion of adiponectin, as well as disordered lipid metabolism in rat model of IR; and the expression level of adipoR1 protein in skeletal muscle is upregulated. These improvements in IR were observed associated with significant reduction of fat accumulation in skeletal muscle along with activation of the AMPK/ACC signaling pathway. In conclusion, EA is evidenced to be able to prevent disordered lipid metabolism and to ameliorate insulin sensitivity. Therefore, EA may represent a promising therapeutic strategy for diseases with obese related to insulin resistance.

## Data Availability

The data used to support the findings of this study are available from the corresponding author upon request.
